# Integrating Artificial Intelligence Into Telemedicine: Evidence, Challenges, and Future Directions

**DOI:** 10.7759/cureus.90829

**Published:** 2025-08-23

**Authors:** Martina Rossi, Shajeel Rehman

**Affiliations:** 1 Research, Private, Strasbourg, FRA; 2 Occupational Health, The State University of New York (SUNY) Farmingdale State College (FSC), New York City, USA

**Keywords:** algorithmic bias, artificial intelligence, data privacy, digital health, machine learning, remote healthcare, telemedicine, wearable technology

## Abstract

Telemedicine has revolutionized healthcare by enabling remote diagnosis, monitoring, and treatment. However, challenges such as clinician workload, data variability, and technological disparities hinder its full potential. Artificial intelligence (AI) offers solutions by automating diagnostics, predictive analytics, and real-time monitoring, yet its integration into telemedicine presents ethical, regulatory, and implementation challenges.

This review explores the role of AI in telemedicine, identifying key applications, challenges, and future directions. A systematic literature search was conducted in PubMed and the Cochrane Library, covering the period from 2015 to 2024, adhering to PRISMA guidelines. Of the 40 identified articles, 31 met the inclusion criteria for thematic evaluation. Relevant studies were selected based on predefined criteria, and thematic evaluation identified trends, barriers, and innovations in AI-driven telemedicine.

AI has been successfully implemented in diverse telemedicine applications. In dermatology, AI-driven image analysis achieves diagnostic accuracy comparable to experts. Ophthalmology benefits from AI-enhanced screening for diabetic retinopathy and glaucoma. AI-powered chatbots and digital assistants improve mental health support and patient triage. Wearable devices utilizing AI facilitate continuous monitoring of cardiovascular and respiratory conditions. Emerging technologies such as blockchain-based digital pathology and decentralized AI models have been proposed, and in some cases demonstrated in proof-of-concept studies, to enhance data protection and accessibility in telemedicine. However, challenges persist, including algorithmic bias, data privacy concerns, regulatory inconsistencies, and limited real-world validation of AI models.

Overall, evidence from multiple specialties indicates that AI can enhance telemedicine by improving diagnostic accuracy, patient monitoring, and remote healthcare delivery. However, the degree of benefit varies across clinical domains, and most studies remain limited in real-world validation. Moreover, ethical considerations, regulatory compliance, and model generalizability require further research. Addressing these gaps will ensure equitable, effective, and scalable AI-driven telemedicine solutions. Future efforts should focus on improving interoperability, standardizing guidelines, and integrating privacy-preserving AI models to facilitate widespread adoption.

## Introduction and background

Telemedicine has become an essential tool in modern healthcare, revolutionizing the way medical services are delivered across the globe [1,2]. It enables remote consultations, disease diagnosis, patient monitoring, and treatment via digital communication technologies, significantly improving healthcare accessibility, specifically in underserved regions [3]. The World Health Organization (WHO) defines telemedicine as the provision of remote healthcare services by healthcare professionals through information and communication technologies for diagnosis, treatment, and prevention of diseases, as well as health promotion [4]. Over the past two decades, telemedicine has experienced significant growth, driven by advances in digital health technologies, the increasing burden of chronic diseases, and the need for more efficient healthcare delivery methods [1,5]. The COVID-19 pandemic further supported its adoption, as global healthcare systems faced unprecedented challenges that required a shift from in-person medical care to remote healthcare services [6,7].

While telemedicine offers several advantages, spanning from overcoming geographical boundaries to decreasing healthcare costs and enabling timely interventions, its implementation also presents some challenges [1]. These include technological limitations, disparities in virtual infrastructure, data security concerns, and regulatory compliance that varies across specific regions [1]. A significant limitation of modern telemedicine models is their dependence on human healthcare providers, which is widely recognized in the literature as contributing to increased clinician workload and exacerbating shortages of clinical specialists [8]. Moreover, telemedicine relies heavily on patient-generated data, the quality of which can be affected by issues such as delayed submission, incomplete or missing information, insufficient detail, and potential inaccuracies, all of which can challenge scientific rigor and decision-making [9]. The ability to analyze vast amounts of complex healthcare data in real time remains a significant challenge in maximizing telemedicine’s full potential [10].

Artificial intelligence (AI) has emerged as a promising strategy to address many of these challenges and enhance the effectiveness of telemedicine. AI, particularly through machine learning and deep learning, facilitates automation of medical image interpretation, predictive analytics for disease progression, real-time patient monitoring, and decision support systems [11,12]. Applications of AI in telemedicine have demonstrated significant potential across diverse medical specialties. For instance, AI-driven computer vision algorithms have been successfully integrated into dermatology and ophthalmology, achieving high sensitivity and specificity for conditions such as melanoma and diabetic retinopathy, with performance in some studies comparable to or exceeding that of human specialists [13-18]. Furthermore, AI-powered chatbots and digital assistants are being used to triage patients, provide mental health support, and streamline teleconsultations. While many of these applications are still in pilot or early-stage deployment, some have undergone clinical validation. For example, conversational agents for mental health have shown improvements in patient engagement and symptom monitoring in randomized controlled trials, and AI-driven triage assistants have demonstrated high accuracy in directing patients to appropriate levels of care in observational studies [19-21]. Wearable devices combined with AI allow for continuous tracking of vital signs, enhancing early detection of conditions such as cardiovascular and respiratory diseases [22-24]. Evidence from clinical studies shows that devices like the Apple Watch and KardiaMobile can detect atrial fibrillation with high sensitivity and specificity. Meanwhile, wearable blood pressure monitors can identify masked or white-coat hypertension, enabling timely intervention [23]. Similarly, pulse oximetry-equipped wearables can detect sleep apnea events, which are linked to increased stroke and cardiovascular risk, facilitating earlier diagnosis and treatment [22].

Despite these improvements, AI-driven telemedicine is still in its early stages, and significant gaps remain in our understanding of its broader impact on healthcare. Many studies have explored AI applications in unique fields; however, comprehensive evaluations of AI’s integration into telemedicine as a whole remain limited, with recent systematic reviews identifying fewer than a dozen studies assessing end-to-end implementation across multiple specialties. Critical issues such as ethical considerations, algorithmic bias, data privacy, regulatory approval, and interoperability between AI-driven telemedicine platforms remain unresolved, with data privacy, algorithmic transparency, and regulatory clarity emerging as the most frequently cited challenges in the literature [25,26]. Moreover, the effectiveness of AI models in real-world clinical settings remains contested, with key points of debate including the paucity of prospective, real-world trials; limited generalizability across diverse patient populations; and the potential for over-reliance by clinicians on opaque algorithmic outputs [27,28].

Given the rapid advancement in AI and its growing integration into telemedicine, a systematic evaluation of the current state of research is essential. This systematic review focuses on peer-reviewed studies published between 2015 and 2024, examining AI modalities such as machine learning, deep learning, natural language processing, and computer vision applied within telemedicine contexts across multiple clinical specialties, including dermatology, ophthalmology, cardiology, mental health, and chronic disease management. The scope is global, encompassing studies from diverse healthcare systems. The review aims to analyze AI applications in telemedicine, identify key challenges and limitations, and explore future directions for optimizing AI-driven healthcare solutions. By synthesizing the current literature, this review offers insights into how AI is shaping telemedicine, the obstacles hindering its full-scale adoption, and potential strategies for overcoming those barriers to ensure ethical, effective, and equitable AI implementation in remote healthcare.

## Review

Methodology

Search Strategy

This review article was guided by the Preferred Reporting Items for Systematic Reviews and Meta-Analyses (PRISMA) reporting guidelines [29]. Comprehensive search strategies were carried out across two main databases: PubMed and Cochrane Library. These databases were selected due to their strong relevance to biomedical and clinical research, particularly in evidence-based medicine and digital health studies.

The following Boolean query was used to find relevant articles:

For PubMed: ("Artificial Intelligence" OR "AI") AND ("Telemedicine" OR "Remote Healthcare") AND ("Machine Learning" OR "Deep Learning" OR "Clinical Imaging" OR "Predictive Analytics") AND ("Ethics" OR "Data Privacy" OR "Legal" OR "Infrastructure" OR "Policy Development" OR "Education Programs" OR "Global Collaboration") AND ("2015"[Date - Publication]: "2024"[Date - Publication]) AND "English"[Language].

For Cochrane Library: ("Artificial Intelligence" OR "AI") AND ("Telemedicine" OR "Remote Healthcare") AND ("Machine Learning" OR "Deep Learning" OR "Clinical Imaging" OR "Predictive Analytics") AND ("Ethics" OR "Data Privacy" OR "Legal" OR "Infrastructure" OR "Policy Development" OR "Education Programs" OR "Global Collaboration").

Filters for the Cochrane Library search included publication year (2015-2024) and language (English). To ensure the relevance of the search strategy and minimize irrelevant results, iterative refinement and pilot testing of search terms were conducted.

Inclusion and Exclusion Criteria

The inclusion and exclusion criteria were carefully designed to ensure the selection of high-quality, relevant studies that provide a comprehensive overview of AI integration in telemedicine. The following criteria guided the methodical screening and selection process:

The inclusion criteria consisted of studies published between 2015 and 2024 to capture developments over the most recent ten-year period. Eligible studies specifically addressed AI applications in human healthcare with a focus on telemedicine, including both empirical evaluations and theoretical discussions covering areas such as machine learning, deep learning, clinical imaging, predictive analytics, and wearable devices. Only studies that met predefined methodological rigor, such as clearly stated objectives, appropriate validation methods, adequate sample sizes, and transparent statistical reporting, were included. Research addressing ethics, data privacy, legal frameworks, infrastructure, policy creation, educational programs, and international collaboration, whether presented as empirical studies or conceptual analyses, was also considered. Furthermore, only peer-reviewed journal articles, systematic reviews, and meta-analyses written in English were included.

The exclusion criteria were as follows: studies not related to AI integration in telemedicine or those focusing on non-healthcare or non-human applications, including both empirical and theoretical articles, lacking a specific focus on AI in telemedicine; gray literature such as conference abstracts, editorials, and opinion articles; articles written in languages other than English; duplicate studies identified during database searches were removed manually; and case studies or prospective studies that were not included in larger meta-analyses or systematic reviews due to their poor generalizability.

Data Extraction and Management

The study selection procedure was carried out in two steps. First, a title and abstract screening was performed using a standardized screening checklist to ensure consistent application of the inclusion and exclusion criteria. Articles that passed the initial assessment underwent a full-text examination to confirm they met all requirements. Data extraction was conducted systematically using Zotero (Corporation for Digital Scholarship (CDS), Fairfax, VA, USA) for reference management and an Excel data extraction table (Microsoft Corp., Redmond, WA, USA) to capture structured study variables. Important information about each selected study was collected, including the authors, year of publication, study aims, methodology, key findings, and thematic focus. To reduce bias, two researchers conducted the whole evaluation procedure, including data extraction, independently. Any disagreements in study evaluations were settled through discussion, with the primary investigator making the final decision. These data were organized into a structured table to aid thematic analysis and ensure thorough coverage of key findings. The extraction process ensured that essential details from each study were documented in a manner that allowed for seamless synthesis and interpretation.

Review Framework

**Figure 1:**
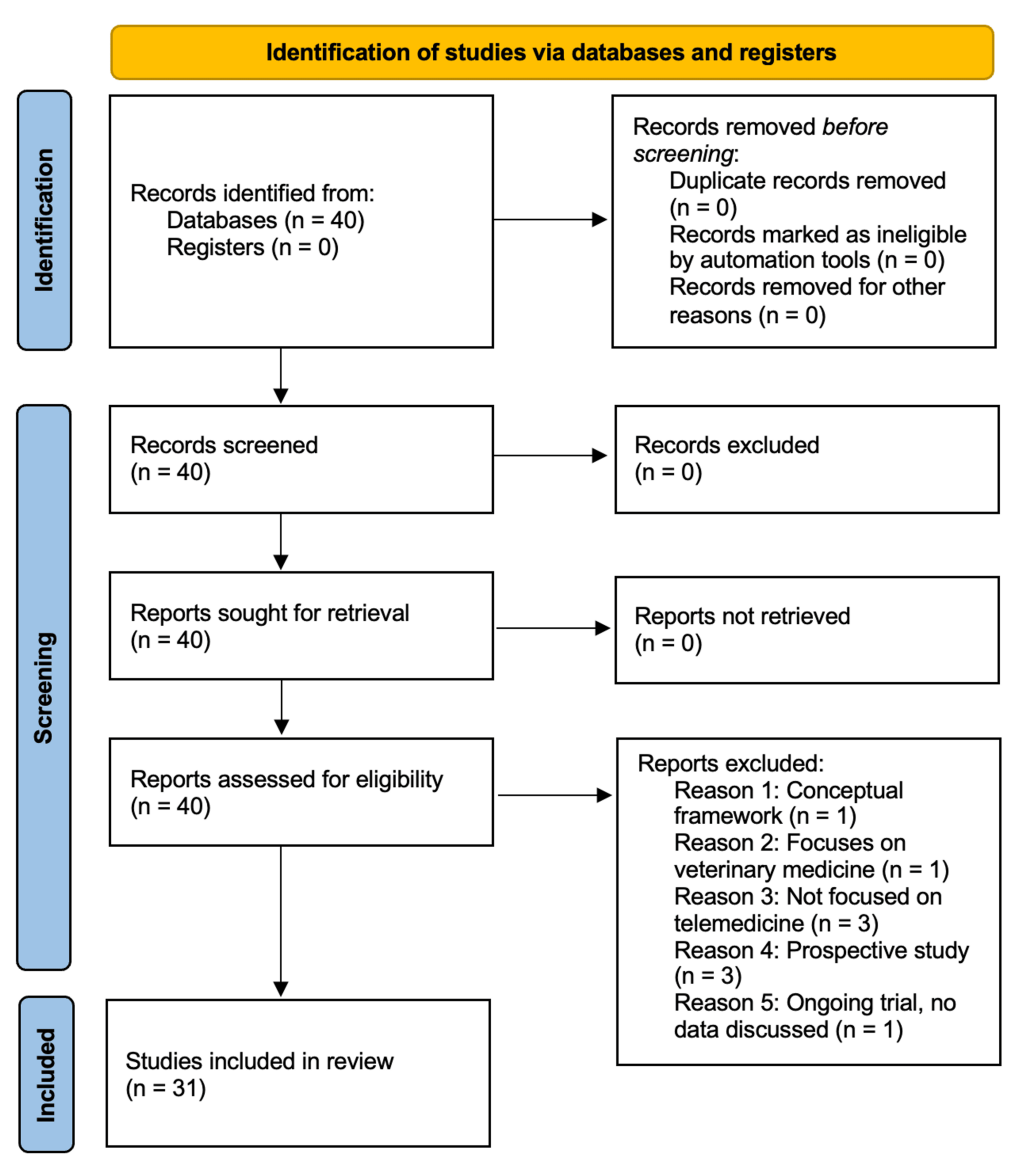
PRISMA 2020 flow diagram for selection of studies retrieved from databases to be included into the systematic review PRISMA: Preferred Reporting Items for Systematic Reviews and Meta-Analyses

A PRISMA flow diagram was used to document each step of the study selection process, including the number of articles retrieved, screened, excluded (with reasons), and eventually included in the review (Figure 1).

Analysis

A thematic analysis was conducted to identify major trends, challenges, and solutions for AI integration in telemedicine. Themes were developed inductively after full-text review and structured categorization of extracted data into an Excel table. The process was based on consensus between the two independent reviewers, who collaboratively grouped studies under thematic categories. The findings were organized into three broad categories: AI applications in telemedicine (e.g., clinical imaging and remote patient monitoring), challenges (e.g., data privacy, regulatory gaps, infrastructure), and proposed solutions (e.g., policy recommendations, healthcare training). Descriptive quantitative data, such as frequency of theme occurrence or study distribution across application areas, were synthesized and presented in tables to aid visualization and highlight key patterns.

Results

This systematic review investigated the implementation of AI in telemedicine across a variety of medical domains, with findings organized by clinical area and thematic outcomes, thereby highlighting advances, persistent challenges, and potential future directions. The search strategy implemented yielded a PubMed search result of 38 articles and a Cochrane Library search result of two articles. Articles not meeting the inclusion criteria were excluded, leaving 31 for review. The studies examined reported how AI-powered tools may enhance remote healthcare delivery by supporting improvements in diagnosis, monitoring, and treatment. To provide a concise summary, Table 1 summarizes the main findings, applications, and technological contributions of the selected studies.

**Table 1:** Summary of selected studies on AI applications in telemedicine highlighting major topics covered AI: artificial intelligence, VR: virtual reality, IoT: Internet of Things, NFT: non-fungible token, FDA: Food and Drug Administration, OCT: optical coherence tomography, IDx-DR: IDx-diabetic retinopathy, AUROC: area under the receiver operating characteristic curve, mHealth: mobile health, eHealth: electronic health, 5G: fifth-generation mobile network, 6G: sixth-generation mobile network, QoS: quality of service, MLP: multi-layer perceptron, COVID: coronavirus disease

Title	Authors	Year	Major topic covered
Artificial intelligence in dermatology: a primer	Young et al. [13]	2020	AI in teledermatology is primarily used for triage and referral to dermatologists, with studies showing diagnostic accuracy comparable to experts. However, real-world clinical validation is lacking, and challenges such as performance assessment, interpretability, and ethical considerations need to be addressed for wider adoption.
Teledermatology and its current perspective	Pasquali et al. [14]	2020	Teledermatology is a key telemedicine application used for consultations, second opinions, education, and monitoring, facilitated by electronic communication tools. Key topics include its basic concepts, the integration of noninvasive imaging and AI, and ethical and legal considerations.
The potential application of artificial intelligence for diagnosis and management of glaucoma in adults	Campbell et al. [30]	2020	AI shows promise in glaucoma detection and management, with machine and deep learning algorithms matching or surpassing human experts. Its potential applications in telemedicine include virtual review clinics and second-opinion support, though concerns remain about clinician deskilling and the need for external validation of AI models.
Applications of artificial intelligence in myopia: current and future directions	Zhang et al. [15]	2022	AI is increasingly used in myopia detection, diagnosis, progression prediction, and treatment through telemedicine platforms and imaging techniques like fundus photography and OCT. Challenges remain in dataset standardization, user acceptance, and ethical and regulatory issues, but AI also shows promise in behavioral interventions using wearable devices.
Artificial intelligence and digital solutions for myopia	Li et al. [16]	2023	AI and digital technologies offer solutions for myopia screening, risk stratification, and progression prediction, aiding both early detection and intervention. Innovations like multimodal AI, explainable AI, and wearable devices enhance accessibility and monitoring, though challenges remain in infrastructure, data management, and clinical validation.
The application of artificial intelligence in diabetic retinopathy: progress and prospects	Xu et al. [17]	2024	AI, particularly deep learning, has advanced diabetic retinopathy detection from binary diagnosis to severity assessment, improving early detection and treatment. With FDA-approved models like IDx-DR, AI is increasingly integrated into telemedicine to address healthcare disparities, though challenges remain in bias, transparency, and ethics.
Diabetic retinopathy screening in the emerging era of artificial intelligence	Grauslund [18]	2022	AI-driven deep learning models improve diabetic retinopathy screening by automating image analysis, enhancing diagnostic accuracy, and reducing healthcare burdens. While regulatory-approved algorithms and telemedicine-based screening programs show promise, challenges remain in clinical implementation, legal regulations, and software integration with national screening systems.
Prospective, longitudinal pilot study: daily self-imaging with patient-operated home OCT in neovascular age-related macular degeneration	Keenan et al. [31]	2021	Home OCT telemedicine systems enable daily self-imaging for neovascular age-related macular degeneration, with automated AI analysis showing high agreement with human grading. This approach allows personalized disease monitoring, potentially optimizing retreatment decisions while reducing unnecessary clinic visits and injections.
Industry 4.0 technologies in maternal health care: bibliometric analysis and research agenda	Sibanda et al. [32]	2024	Industry 4.0 technologies, including AI, telemedicine, and wearable devices, are transforming maternal healthcare by optimizing care processes, treatment methods, and automated pregnancy monitoring. Key research focuses on risk prediction, digital health, and self-care, though challenges remain in governance, adoption, infrastructure, and data security.
Revolutionizing maternal health: The role of artificial intelligence in enhancing care and accessibility	Mapari et al. [33]	2024	AI is transforming maternal healthcare by improving early complication detection, personalized care, and remote monitoring, particularly benefiting underserved areas through telemedicine. While AI enhances diagnostic accuracy and intervention timing, challenges like data privacy and algorithmic bias must be addressed to ensure ethical and equitable integration.
Revolutionizing oncology: a comprehensive review of digital health applications	Borkar et al. [34]	2024	Digital health is reshaping oncology by improving early detection, personalized treatment, and patient care through AI, predictive analytics, and virtual technologies. While these innovations enhance efficiency and accessibility, challenges like data privacy and equitable access must be addressed to ensure ethical implementation and patient-centered progress.
Tele-robotics and artificial-intelligence in stroke care	Rabinovich et al. [35]	2020	Robotic surgical systems and AI-driven technologies are transforming cerebrovascular procedures, offering precision and accessibility improvements. However, challenges such as limited clinical experience, ethical concerns, and regulatory barriers hinder widespread adoption. Ongoing research in AI and tele-robotics may unlock new possibilities for neurovascular surgery, enhancing patient outcomes.
An artificial intelligence platform for movement analysis and rehabilitation: clinical applications of stepsense to complex pain and long COVID	Pelah et al. [36]	2021	The StepSense platform leverages AI-driven gait analysis and VR biofeedback to aid rehabilitation in complex pain and long COVID. Clinical trials suggest improved mobility and patient satisfaction. With long COVID affecting millions, AI-based solutions like StepSense offer innovative approaches for assessment and therapy.
Health data space nodes for privacy-preserving linkage of medical data to support collaborative secondary analyses	Baumgartner et al. [22]	2024	The Health Data Space node infrastructure enables privacy-preserving linkage of medical data to support secondary use in healthcare. A real-world implementation in a heart failure registry demonstrates its feasibility, aggregating over 5.2 million data points. This initiative aligns with the European Health Data Space vision, fostering secure, collaborative health data analysis.
Improving diagnosis through digital pathology: proof-of-concept implementation using smart contracts and decentralized file storage	Subramanian and Subramanian [37]	2022	A blockchain-based digital pathology system leveraging NFT smart contracts and decentralized storage addresses cost, security, and data transmission challenges. By separating metadata from file storage, the prototype enhances accessibility, privacy, and AI-driven diagnosis, paving the way for more efficient and scalable digital pathology solutions.
Mobile diagnostic clinics	Baron and Haick [38]	2024	AI-driven mobile diagnostic clinics enhance accessibility to healthcare by integrating advanced technologies for real-time screening, diagnosis, and continuous monitoring. Key challenges include data privacy, system integration, and ensuring ethical, bias-free AI implementation.
Aeye: a deep learning system for video nystagmus detection	Wagle et al. [39]	2022	A deep-learning model (aEYE) was developed to detect nystagmus in video-oculography recordings, achieving an AUROC of 0.86 with 88.4% sensitivity and 74.2% specificity. Performance remained stable with lower image resolutions but declined with reduced sampling rates, highlighting its potential for automated neuro-ophthalmic diagnosis.
The impact of artificial intelligence on optimizing diagnosis and treatment. Plans for rare genetic disorders	Abdallah et al. [40]	2023	AI and machine learning enhance the diagnosis and treatment of rare genetic disorders by enabling early detection, personalized therapies, and drug discovery. Key advancements include improved data analysis, patient-centric care, and ethical considerations in AI-driven healthcare.
Combining mobile-health (mHealth) and artificial intelligence (AI) methods to avoid suicide attempts: the Smartcrises study protocol	Berrouiguet et al. [19]	2019	Smartcrisis leverages smartphone sensors, wearable technology, and AI-driven signal processing to assess suicide risk based on changes in sleep and appetite. Real-time digital footprint for mental health monitoring was analyzed while addressing ethical and security concerns.
Posture estimation model combined with machine learning estimates the radial abduction angle of the thumb with high accuracy	Shinohara et al. [20]	2024	Combining MediaPipe-Hands with machine learning significantly improves the accuracy of thumb radial abduction angle estimation from video images. This system enables reliable, contactless functional assessment, with potential applications in telemedicine and rehabilitation.
Enhancing healthcare through sensor-enabled digital twins in smart environments: a comprehensive analysis	Adibi et al. [21]	2024	Sensor-driven digital twin technology enhances healthcare in smart environments by integrating IoT, AI, and wearable health devices for real-time monitoring and telemedicine. This approach improves personalized care, emergency response, and healthcare delivery while addressing key challenges and opportunities.
First do no harm: legal principles regulating the future of artificial intelligence in health care in South Africa	Donnelly [41]	2022	AI-powered medical robots and systems operate with a degree of autonomy, posing legal and ethical challenges in South Africa’s healthcare framework. Key reforms include updating regulations for AI-driven medical devices, modernizing restrictive telemedicine guidelines, and revising liability laws to ensure patient protection and accountability.
Ethical issues in using ambient intelligence in health-care settings	Martinez-Martin et al. [42]	2021	Ambient intelligence in healthcare improves safety and efficiency through contactless sensors and wearable devices that collect and analyze data using machine learning. However, its widespread use raises ethical concerns, including privacy, data management, bias, and informed consent, which must be addressed for broader acceptance.
The crucial role of 5G, 6G, and fiber in robotic telesurgery	Dohler et al. [43]	2024	5G and future 6G networks enable robotic telesurgery by minimizing latency and ensuring data reliability for real-time remote operations. Ultra-low latency and high data transfer rates support critical modalities like kinesthetic, audiovisual, and tactile feedback. Kinesthetic data require particularly low latency for effective surgeon control. Network reliability, Quality-of-Service agreements, and AI-driven predictive analytics in 6G could further enhance telesurgery’s precision, safety, and accessibility, shaping the future of remote surgical care.
Applications of fog computing in healthcare	Jeyaraman et al. [44]	2024	Fog computing enhances real-time healthcare by enabling rapid diagnostics, continuous monitoring, and telemedicine through localized data processing. Addressing interoperability, scalability, and security challenges is key to maximizing its impact.
The challenges of telemedicine in rheumatology	Song et al. [45]	2021	Telemedicine in rheumatology improves access and continuous monitoring through teleconsultations, mobile apps, and AI-driven tools. Challenges include data security, legal issues, reimbursement, and guideline implementation.
Rebirth of distributed AI—a review of eHealth research	Khan and Alkaabi [46]	2021	Federated learning enables AI-driven smart city services by ensuring data privacy and reducing training time. Its application in eHealth requires addressing design goals, technical requirements, and existing challenges.
Digital technologies and data science as health enablers: an outline of appealing promises and compelling ethical, legal, and social challenges	Cordeiro [47]	2021	Digital health technologies, including AI, telemedicine, and wearable devices, promise to transform healthcare but raise ethical, legal, and social challenges. Key concerns include patient autonomy, data security, equity, and the evolving role of human interaction in medicine.
Privacy-preserving automatic collection of acoustic voiding events	Arjona et al. [48]	2023	A low-cost ultrasonic platform with machine learning enables automatic, privacy-preserving uroflowmetry at home. The Multi-layer Perceptron classifier achieved 97.8% accuracy, supporting urology telemedicine in resource-limited settings.
Harnessing the power of AI: a comprehensive review of its impact and challenges in nursing science and healthcare	Yelne et al. [49]	2023	AI is transforming nursing and healthcare through personalized care, diagnostics, and predictive analytics, but challenges like data privacy, ethics, and algorithmic bias remain. To harness AI’s full potential while preserving human-centered care, interdisciplinary collaboration and ethical frameworks are essential.
The economic impact of artificial intelligence in health care: systematic review	Wolff et al. [50]	2020	Despite AI’s growing role in healthcare, few studies rigorously assess its economic impact, and existing analyses suffer from methodological shortcomings. Future research must incorporate comprehensive cost-effectiveness evaluations, including initial investments, operational costs, and viable alternatives, to support informed decision-making in AI adoption.

AI and Telemedicine Integrations Across Medical Specialties

**Figure 2:**
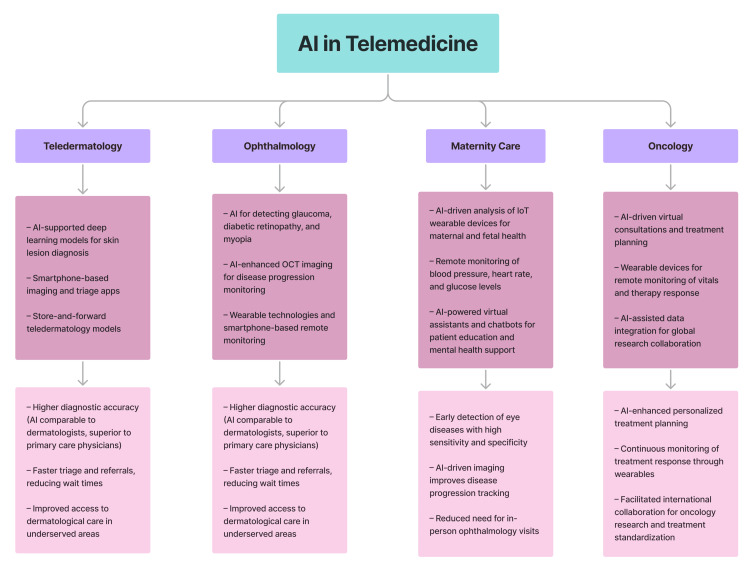
AI applications in telemedicine across medical specialties. AI enhances diagnosis, treatment planning, and remote monitoring in teledermatology, ophthalmology, maternity care, and oncology AI: artificial intelligence, OCT: optical coherence tomography Created using Figma (Figma, Inc., San Francisco, CA, USA)

Teledermatology: Teledermatology was one of the first fields in healthcare to integrate AI and telemedicine, owing to the image-based nature of dermatological diagnosis and increasing service demand (Figure 2) [13,14]. In teledermatology contexts, AI-supported deep learning models achieved diagnostic accuracy comparable to dermatologists (0.67 vs. 0.63) and outperformed primary care physicians (0.45) in identifying 26 skin conditions [13]. Smartphone apps for skin lesion imaging and triage are available; however, empirical evaluations have shown that their real-world diagnostic accuracy remains limited [13]. AI-enhanced teledermatology has the potential to improve access to care in underserved and rural locations by expediting referrals and reducing wait times. However, disparities in training data representation, particularly the underrepresentation of darker skin tones, may limit diagnostic accuracy in these populations, potentially exacerbating health inequities [13]. The asynchronous store-and-forward model, in which clinical and dermoscopic images are transmitted for deferred specialist review, has shown particular utility in such situations. In particular, it has been observed that adding dermoscopic images significantly improved diagnostic sensitivity (from 86.57% to 92.86%) and specificity (from 72.33% to 96.24%) compared to clinical images alone [14].

Ophthalmology: AI has shown great promise in ophthalmology, particularly in tasks such as automated image analysis, risk stratification, disease progression prediction, and self-monitoring, with applications spanning myopia, diabetic retinopathy, glaucoma, and age-related macular degeneration (Figure 2) [15-18,30,31]. Across several studies, AI systems demonstrated high diagnostic accuracy for a wide range of conditions, particularly in image-based tasks like retinal fluid detection and classification of disease stages. Reported sensitivity and specificity in controlled studies often exceeded 90%. In some cases, AI performance has matched or surpassed that of human specialists, though most evaluations remain limited to internal validation or retrospective comparisons [15-18,30]. Advanced imaging modalities such as optical coherence tomography (OCT) and OCT angiography have enhanced AI’s predictive capabilities. For example, in glaucoma, a deep learning model predicted future Humphrey visual field mean deviation values up to 5.5 years in advance from a single visual field test, achieving a high correlation (r = 0.92) between predicted and actual values in a real-world dataset [30]. Telemedicine solutions powered by AI allow for remote screening, triaging, and monitoring, particularly in underserved areas [15,17,18,30]. Wearable technologies such as Clouclip and smartphone-based apps have enabled remote monitoring, resulting in actionable insights into visual habits and disease progression [15-17]. Home OCT systems for retinal diseases have also demonstrated significant agreement with expert assessments for fluid detection and treatment scheduling [31].

Maternity care: Telemedicine has also demonstrated its potential to revolutionize maternity healthcare using Industry 4.0 technologies (Figure 2) [32]. Telehealth platforms, along with wearable Internet of Things (IoT) devices, allow for remote monitoring of maternal and fetal health parameters like blood pressure and heart rate, eliminating the need for frequent clinic visits and enhancing access in underprivileged areas [32]. Furthermore, pilot and observational studies have shown that AI enhances telemedicine by analyzing real-time data from wearable blood pressure and heart rate monitors, continuous glucose sensors, and uterine-contraction devices to detect early signs of preeclampsia, gestational diabetes, and preterm labor, thereby enabling timelier interventions and more personalized care plans [33]. Virtual assistants and AI-powered chatbots promote self-care by providing personalized advice, reminders, and mental health support, especially for diseases such as postpartum depression [33]. Furthermore, cloud-based telehealth solutions improve collaboration among healthcare professionals by securely storing and sharing medical records in real time [32,33].

Oncology: Telemedicine has also shown promise in oncology, allowing for remote consultations, follow-ups, and mental health treatment while minimizing the need for in-person visits (Figure 2) [34]. AI-powered virtual care offers personalized diagnosis and treatment planning, with the potential to improve patient outcomes. Moreover, wearable devices allow for remote monitoring of vital signs and treatment responses, supporting proactive care. With the patients’ data obtained, international collaboration can generate comprehensive datasets to advance research and treatment standardization. These technologies can boost cancer knowledge access, improve treatment coordination, and reduce healthcare costs [34].

Emerging Technologies and Innovations in Telemedicine

**Figure 3:**
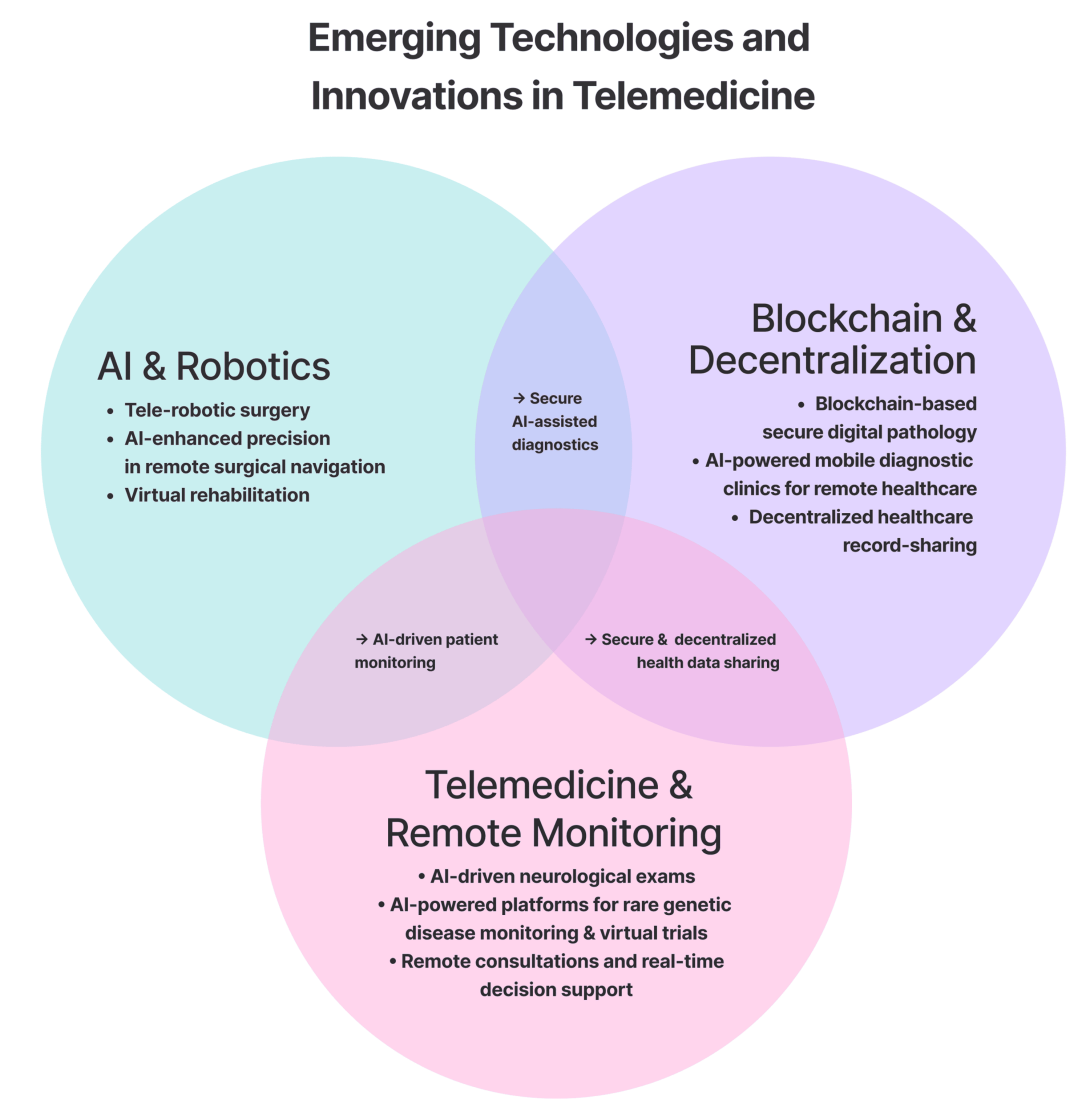
Emerging technologies and innovations in telemedicine, including AI and robotics, blockchain and decentralization, and telemedicine and remote monitoring AI: artificial intelligence Created using Figma (Figma, Inc., San Francisco, CA, USA)

Robotics and virtual rehabilitation: Telemedicine, aided by AI-powered robotics and virtual rehabilitation platforms, is emerging as a promising approach in stroke treatment and movement analysis, particularly in early-stage research and pilot applications (Figure 3). Tele-robotic systems such as CorPath GRX demonstrate the technical feasibility of remote endovascular manipulation in stroke care and show promise for improving accessibility in underserved areas [35]. AI integration streamlines surgical navigation and improves procedure precision, bringing specialized care to remote locations. Meanwhile, technologies like StepSense provide virtual rehabilitation solutions via virtual reality (VR) biofeedback, enabling remote gait analysis and therapy for patients with chronic pain and long COVID. Preliminary data from a small-scale, single-site controlled trial (n = 10) suggest that VR-based interventions were associated with greater improvements in mobility, as measured by the six-minute walk test, and higher patient satisfaction compared to standard care [36].

Blockchain and decentralized solutions: The integration of blockchain, AI, and decentralized technologies has led to substantial breakthroughs in telemedicine (Figure 3). One study outlined a conceptual blockchain-based architecture for secure and scalable digital pathology using ERC-721 non-fungible tokens and the InterPlanetary File System to facilitate remote diagnostic image exchange, with the proposed benefit of reducing infrastructure costs [37]. This method also enabled AI-assisted diagnosis of cancer, urology, and gastrointestinal illnesses. Another innovation, mobile diagnostic clinics, has been proposed as a concept that integrates AI and portable diagnostic instruments to deliver telemedicine services, such as remote consultations and real-time decision support, to improve access to care and early disease detection in underserved areas [38]. Furthermore, aEYE, a deep learning system for video-based nystagmus detection, was evaluated on 435 monocular infrared video-oculography recordings from 30 patients with acute dizziness and vertigo, achieving an AUROC of 0.86, a sensitivity of 88.4%, and a specificity of 74.2% in 60 Hz recordings, with minimal performance loss at lower image resolutions, with results obtained via threefold cross-validation [39]. Telemedicine for rare genetic diseases has demonstrated the ability of AI-powered platforms to aid in early diagnosis, remote patient monitoring, and virtual clinical trial recruitment [40]. These innovations highlight how decentralized solutions, combined with AI, improve accessibility to healthcare while reducing infrastructure requirements and associated costs.

Data Integration and Privacy in AI Telemedicine

**Figure 4:**
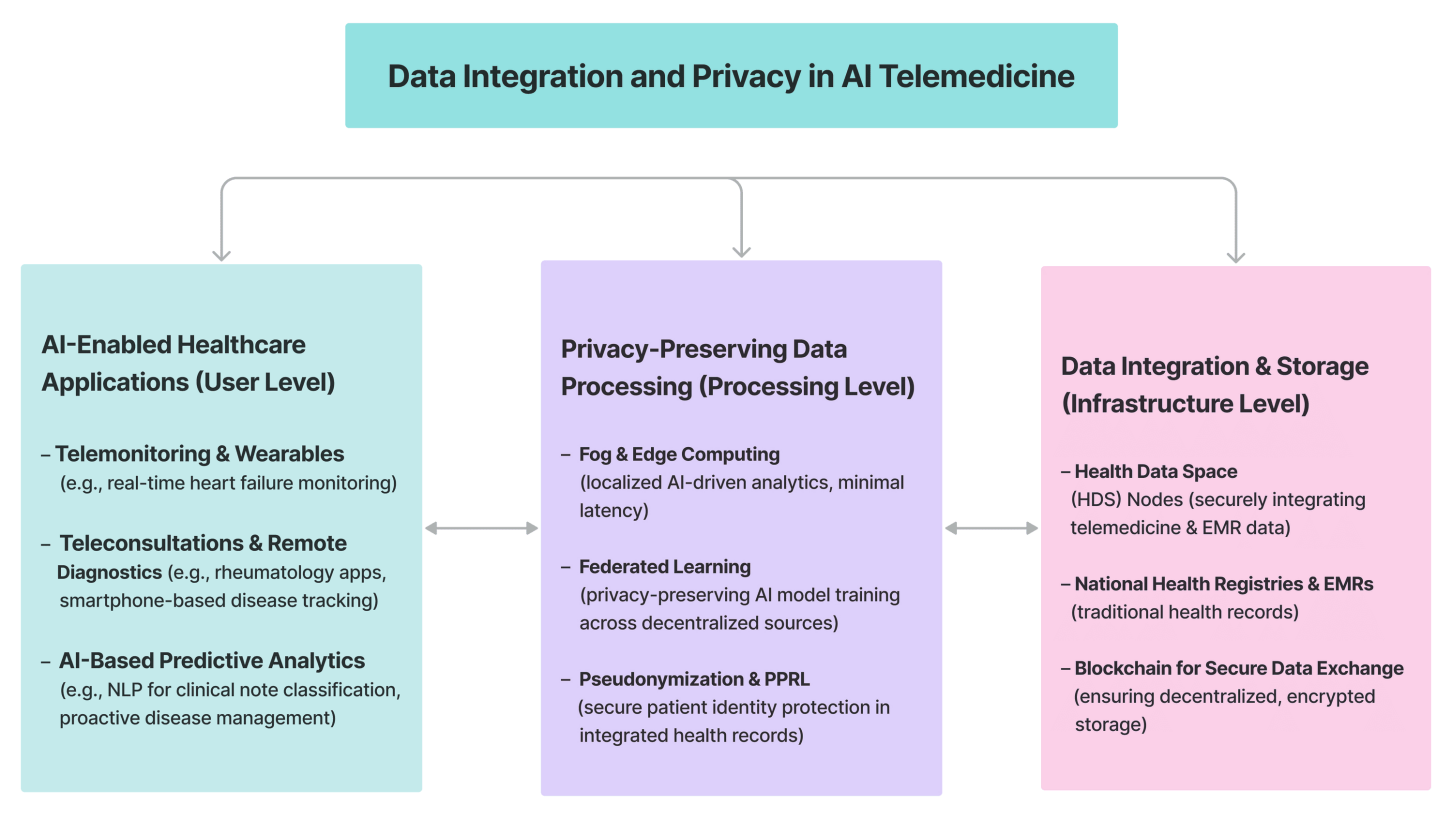
Key components of data integration and privacy in AI telemedicine, including AI-enabled healthcare applications (user level), privacy-preserving data processing (processing level), and data integration and storage (infrastructure level) AI: artificial intelligence, NLP: natural language processing, PPRL: privacy-preserving record linkage, HDS: Health Data Space, EMRs: electronic medical records

Health data management solution: The creation of Health Data Space (HDS) nodes is a big step forward in combining telemedicine data with traditional clinical sources, such as electronic medical records and national health registries (Figure 4). In a proof-of-concept study with heart failure patients, HDS nodes securely integrated data from telemonitoring devices and other sources while protecting patient privacy using approaches such as pseudonymization and privacy-preserving record linkage. The system demonstrated scalability by integrating data from 5,004 patients, including over 2.9 million clinical free-text notes and 5.2 million clinical events. It achieved processing rates of up to 1,151 data points per second in extract, transform, and load operations [22]. It supported machine learning applications such as natural language processing-based clinical note classification and highlighted the role of telehealth data in comprehensive patient analyses and quality-of-life assessments.

Privacy-preserving techniques: Another AI-driven telemedicine innovation is the integration of fog computing, which, through localized data processing, enables real-time diagnostics, intraoperative monitoring, and remote patient care through localized data processing that protects privacy and minimizes latency (Figure 4) [44]. These capabilities are supported by empirical data from simulation-based evaluations that highlight their technical feasibility and potential impact on healthcare delivery [44]. Moreover, this technique is supplemented by AI-powered predictive analytics for proactive disease management. In the field of rheumatology, it has been shown that teleconsultations, smartphone applications, and wearable devices improve disease monitoring and minimize travel burdens. However, privacy concerns and inadequate app validation persist [45]. Furthermore, another study based on simulation studies and conceptual models explored how federated learning and edge computing enable real-time remote diagnostics and continuous patient monitoring while protecting data privacy [46].

AI-Driven Mental Health and Rehabilitation Solution

**Figure 5:**
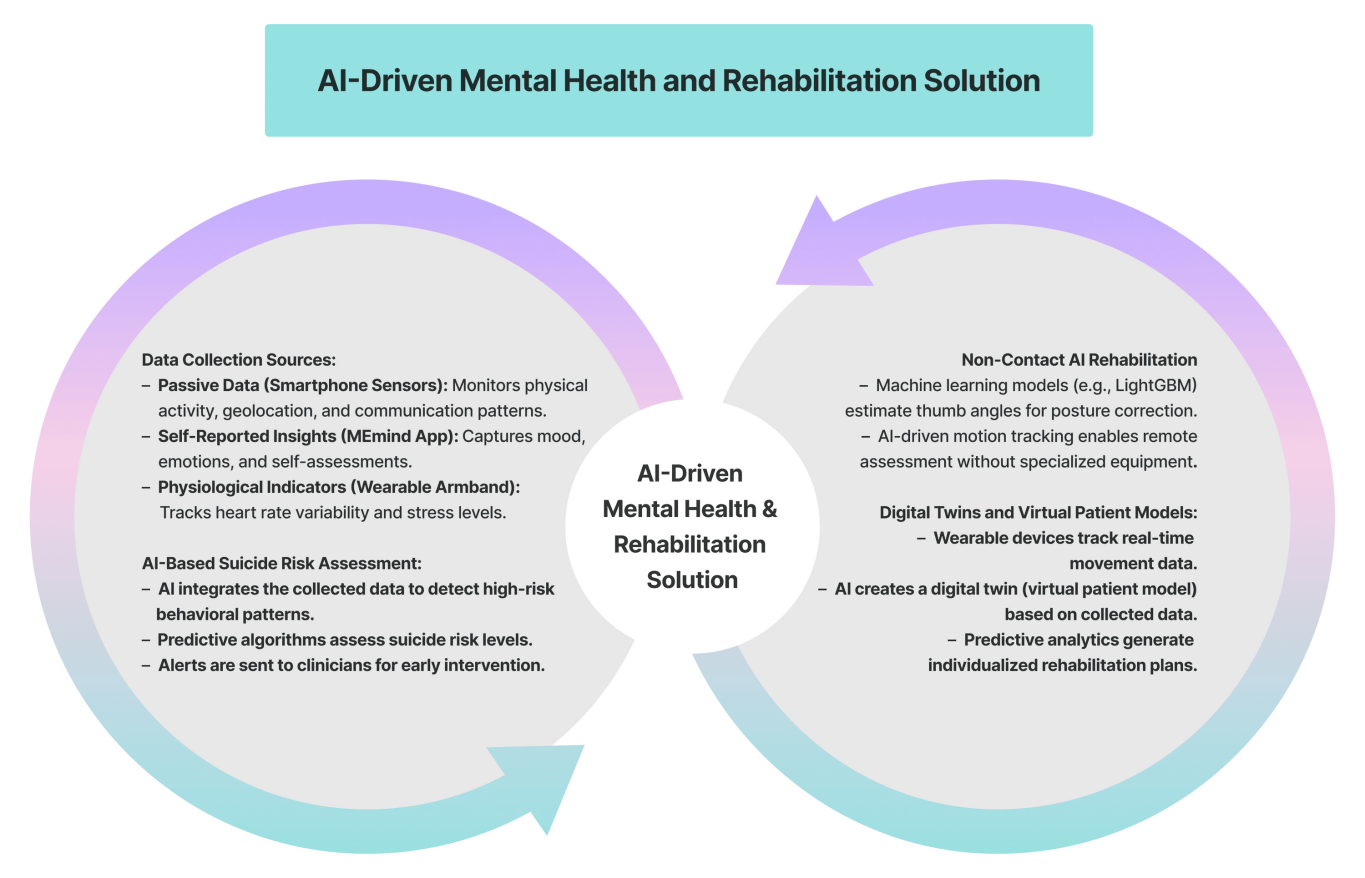
AI-driven mental health and rehabilitation solutions integrate data from smartphone sensors, wearable devices, and self-reported insights to assess behavioral patterns and suicide risk. AI-powered rehabilitation utilizes machine learning for posture correction, motion tracking for remote assessments, and digital twin models to generate personalized treatment plans AI: artificial intelligence Created using Figma (Figma, Inc., San Francisco, CA, USA)

Suicide prevention and behavioral monitoring: Telemedicine has been shown to enhance mental healthcare by enabling continuous monitoring and early intervention for at-risk individuals (Figure 5). In this context, a prospective multisite clinical trial protocol explored the integration of AI and mobile health (mHealth) in telemedicine to prevent suicide attempts through real-time behavioral monitoring and individualized interventions [19]. By integrating passive data collection via the eB2 app (monitoring physical activity, geolocation, and communication patterns) with self-reported insights via the MEmind app and physiological indicators measured by armbands, a comprehensive assessment of the status of patients is possible. Then, AI-powered programs assess this data to identify suicide risk, directing clinicians toward prompt interventions. These predictive models are being evaluated against standardized clinical assessments as part of an ongoing validation process [19].

Rehabilitation and virtual patient models: Telemedicine developments are also evident in promising research that uses AI for remote rehabilitation and patient monitoring (Figure 5) [20]. Using a simulation-based study with data from 10 healthy volunteers, LightGBM was applied to estimate thumb radial abduction angles with high accuracy (CC = 0.99), offering a cost-effective, non-contact telemedicine solution for posture assessment without requiring specialized equipment [20]. However, further validation in larger and clinically diverse patient populations is necessary before broad application. Similarly, another study described a proof-of-concept framework combining digital twins with wearable devices to construct virtual patient models for real-time monitoring, predictive analytics, and individualized care, with early implementations observed in different settings [21].

Challenges: The use of AI in telemedicine creates several potential challenges across a variety of healthcare applications. While AI-powered telemedicine enables remote diagnostics, decision-making, and personalized care, it also raises concerns in regulatory, technical, and ethical domains. For instance, a recent study highlighted South Africa’s outdated telemedicine guidelines and called for AI-specific legislation, emphasizing the importance of AI in remote consultations and rural care delivery while raising ethical concerns such as informed consent and liability for AI errors [41]. Similarly, another study investigated ambient intelligence for passive remote monitoring, enhancing clinical decision-making with AI-driven insights while highlighting issues related to privacy, bias, and explainability, and proposing mitigation strategies such as robust data governance and encryption, use of diverse and representative datasets with regular algorithm audits, and adoption of interpretable models with transparent communication [42]. Moreover, infrastructure limitations and regulatory barriers in robotic telesurgery have been raised, including cross-border licensing challenges, liability concerns in the event of technical failures, and variations in device approval processes, despite the promise of ultra-low latency networks for AI-assisted robotic surgery and telemedicine [43].

Another challenge stems from biases in AI models trained predominantly on European and East Asian populations, which limits the generalizability of findings to diverse settings. For example, one convolutional neural network achieved an area under the curve of 0.96 on an Asian dataset but dropped to 0.88 on a Caucasian dataset, and commercial systems trained on predominantly White datasets have shown increased error rates for Black individuals, underscoring the need for diverse training data [13,14]. Technical constraints, such as image quality inconsistencies and the lower reliability of AI models on external datasets, further limit implementation [13]. Ethical and legal barriers persist as well, particularly regarding the “black box” nature of AI, which refers to a lack of transparency on how AI models reach diagnostic conclusions [15-18,30,31].

Data privacy concerns remain a major barrier, alongside the lack of standardized reimbursement models for AI-integrated telemedicine [14]. In low-resource settings, particularly in maternal healthcare, obstacles such as high implementation costs, privacy concerns, and algorithmic biases create disparities in access to care [32]. Furthermore, disparities in access to digital health technologies underscore the need for equitable and secure implementation strategies [34].

One more challenge is the lack of interoperability between telemedicine systems, which, along with resource limitations, significantly limits large-scale adoption [46]. Distributed AI architectures and blockchain-based frameworks have been conceptually proposed to address interoperability challenges in telemedicine [46]. Also, while AI-powered predictive analytics and remote monitoring have enhanced healthcare delivery, they have increased the risks of data breaches and algorithmic biases [49].

To tackle such challenges, research has been conducted focusing on the development of privacy-preserving AI platforms. For instance, a recent study demonstrated how non-intrusive, real-time health monitoring can be seamlessly integrated into telemedicine for remote uroflowmetry [48]. Moreover, it is also important to consider that economically, AI-driven telemedicine has the potential to reduce healthcare-associated costs significantly; however, cost-effectiveness analyses to date have been limited to a few domains, leaving most other specialties without a comprehensive economic evaluation [50].

Discussion

The integration of AI in telemedicine has demonstrated considerable promise across different medical specialties, providing improvements in prognosis, patient monitoring, and customized treatment. This systematic review highlighted that AI-powered telemedicine enhances healthcare accessibility, mainly in remote and underserved regions, by facilitating screening and triaging, although few studies reported direct access to metrics.

In dermatology, AI has achieved diagnostic accuracy comparable to dermatologists; one randomized clinical trial demonstrated improved diagnostic performance and cost-effectiveness through the integration of dermoscopic images in teleconsultations, supporting the potential for clinical implementation, though real-world validation remains limited [13,14]. Similarly, ophthalmology has benefited from AI-driven screening and monitoring for conditions such as diabetic retinopathy and glaucoma, with models attaining high sensitivity and specificity [15-18,30]. However, issues persist regarding external validation, dataset diversity, and the potential risk of clinician deskilling, where over-reliance on AI for routine image interpretation may diminish clinicians’ diagnostic proficiency [15,30,31]. In maternal healthcare, AI and telemedicine have facilitated remote monitoring, early problem detection, and customized care, but challenges, which include infrastructure limitations and algorithmic bias, still need to be addressed [32,33]. Oncology applications of AI have expanded patient access to digital care, improving treatment coordination and long-term monitoring [34]. Emerging technologies, such as AI-driven robotic telesurgery and wearable rehabilitation tools, have demonstrated the potential to enhance surgical precision and post-treatment recovery, though regulatory and ethical challenges persist [35,36].

Beyond clinical applications, AI-driven data management systems, such as HDS nodes and federated learning frameworks, have enhanced privacy-preserving medical records integration [22,46]. Nevertheless, interoperability and standardization issues remain key boundaries to widespread adoption. Moreover, AI’s role in mental health monitoring and suicide prevention has emerged as a novel telemedicine application, leveraging real-time behavioral data analysis to support early intervention strategies [19]. Despite these advancements, the challenges identified in this systematic review, including bias in AI models, privacy concerns, and regulatory gaps, underscore the need for robust validation frameworks and policy-driven solutions to ensure equitable and ethical AI use in telemedicine. The development and testing of such frameworks remain a key area for future research.

Practical Application of AI in Telemedicine

The findings of this review suggest that AI-powered telemedicine can significantly improve healthcare delivery by decreasing diagnostic errors, expediting triage, and enhancing remote patient monitoring. While a comprehensive assessment of clinical endpoints was limited, some reviewed studies reported indicators suggestive of improved clinical outcomes [17,18]. For example, FDA-approved AI models for diabetic retinopathy screening have facilitated early intervention, decreasing the risk of disease progression [17]. In stroke care, AI-assisted robot telesurgery has extended treatment accessibility, particularly in geographically isolated areas [35,43]. Healthcare providers can leverage AI to enhance decision-making, optimize workflow efficiency, and customize patient care. However, for AI-driven telemedicine to be widely applied, healthcare institutions must address challenges including clinician education, AI interpretability, and ethical concerns [42]. Ensuring clinician involvement in AI model development and validation may be crucial in promoting trust in AI and fostering its adoption. Moreover, regulatory bodies should establish clear guidelines for AI validation and integration, emphasizing transparency, data security, and equitable access [41]. Telemedicine platforms incorporating AI should also prioritize patient engagement by incorporating explainable AI features, allowing both clinicians and patients to understand AI-driven decisions [31,42]. This is particularly relevant in specialties like oncology and mental health, where patient trust is critical for adherence to treatment plans [19,34]. Furthermore, reimbursement models for AI-powered telemedicine must be standardized to promote wider adoption and financial sustainability [14,48]. Some studies have discussed existing reimbursement structures, such as payment per teleconsultation in national systems like Spain’s or specialist-per-consultation models in the Netherlands, offering early frameworks for cost-effective integration [14].

Future Directions

While AI has proven its potential in telemedicine, several areas warrant additional research and improvement. Clinical validation and generalizability remain essential, with future studies needing to focus on large-scale, multi-center validation of AI models to assess their applicability across diverse populations, a gap notably unaddressed by most reviewed studies. Addressing bias in AI models, mainly those developed predominantly on data from particular geographic areas, is critical to ensure equitable healthcare outcomes [33,49]. Regulatory and ethical frameworks also need to be developed, with standardized guidelines for AI in telemedicine covering data privacy, liability in AI-driven choices, and clinician oversight [41]. Ethical considerations, including informed consent and algorithmic transparency, need to be prioritized to build trust amongst healthcare providers and patients [42,49]. The potential of AI in personalized medicine should be further explored, mainly in chronic disease management and rehabilitation [20,36]. AI-powered predictive analytics can improve individualized treatment plans, enhancing long-term patient adherence and outcomes [20,21,36]. Future research should also focus on interoperability and data integration, with particular attention to federated learning, which has been actively explored for privacy-preserving health applications, as well as blockchain-based health data management and decentralized AI-driven systems. However, these approaches remain at a conceptual or proof-of-concept stage, mainly due to scalability and interoperability challenges, alongside unresolved regulatory and governance issues that prevent clinical implementation [22,46]. Secure and standardized data-sharing protocols will be essential for maximizing AI’s effect on healthcare delivery [22].

Moreover, research on the financial implications of AI-powered telemedicine remains limited. Notably, while some studies offered partial cost-effectiveness evaluations (e.g., projecting savings from workflow efficiency or reduced hospital admissions), no studies included in this review conducted a methodologically complete cost-benefit analysis that accounts for implementation costs, long-term sustainability, and multi-dimensional healthcare outcomes [50]. In this regard, future research should prioritize comprehensive economic evaluations, including implementation costs, operational expenses, and long-term financial sustainability, to support informed decision-making by healthcare policymakers [50]. Given the increasing adoption of AI-driven mental health applications, further research should explore the long-term impact of AI-based interventions on patient outcomes [19]. Ethical considerations surrounding continuous behavioral monitoring and data privacy should also be examined to ensure responsible implementation [19,49]. Finally, the combination of AI with emerging technologies such as 5G, AR, and IoT presents new opportunities for telemedicine [21,43]. Ultimately, dedicated research into these integrations can further enhance real-time remote diagnostics and treatment.

## Conclusions

This systematic evaluation underscores the transformative potential of AI in telemedicine, demonstrating its potential to enhance diagnostic accuracy, remote monitoring, and personalized patient care across various medical domains. AI-powered tools have already demonstrated benefits in dermatology, ophthalmology, mental health, and chronic disease management, particularly in improving diagnostic accuracy, workflow efficiency, and healthcare accessibility. Real-world outcome evidence is relatively stronger in ophthalmology, where FDA-approved AI tools for diabetic retinopathy screening have shown clinical impact, and in dermatology, where randomized trial data support integration into teleconsultations. In contrast, evidence remains preliminary in mental health and chronic disease management, where most studies are pilot or simulation-based, underscoring the need for larger-scale validation. Still, the widespread integration of AI in telemedicine is hindered by critical challenges, including ethical considerations, data privacy concerns, regulatory inconsistencies, and algorithmic bias. The lack of robust real-world validation and standardized guidelines further limits AI’s clinical applicability, as most studies relied on internal evaluations without assessing generalizability to diverse clinical settings. Addressing these barriers requires a multifaceted approach: strengthening interdisciplinary collaboration, implementing clear regulatory frameworks, and ensuring transparent AI development processes. Future advancements in AI-driven telemedicine should prioritize equitable healthcare access, clinician involvement in model validation, and privacy-preserving AI methodologies, such as federated learning and blockchain-based data management.

Additionally, cost-benefit evaluations are crucial for assessing the long-term financial viability of AI integration in healthcare systems. However, as highlighted in this review, no methodologically complete cost-benefit analyses have yet been conducted, underscoring the urgency of future research in this area. To ensure meaningful evidence for policymakers, such studies should include key parameters such as implementation costs, operational expenses, long-term sustainability, and equity impacts. By overcoming these challenges and improving patient outcomes, AI-powered telemedicine has the potential to transform healthcare delivery globally, minimizing inequalities and increasing healthcare efficiency.
